# Diagnostic Stewardship Trends and Antimicrobial Resistance Profiles of Bacteria Isolated in Zambia: A Five-Year Retrospective Study (2020–2024)

**DOI:** 10.3390/antibiotics14111136

**Published:** 2025-11-10

**Authors:** Joseph Yamweka Chizimu, Aubrey Chichonyi Kalungia, Steward Mudenda, Zoran Muhimba, Chileshe Lukwesa, Webrod Mufwambi, Amon Siame, Kelvin Mwangilwa, Priscilla Gardner, Jimmy Hangoma, Victor Daka, Chikwanda Chileshe, Ntombi B. Mudenda, Maisa Kasanga, Misheck Shawa, Herman Chambaro, Duncan Chanda, Taona Sinyawa, Bertha Chibwe, Kaunda Kaunda, Kaunda Yamba, O-Tipo Shikanga, Freddie Masaninga, Mpela Chibi, Sandra Diana Mwadetsa, Doreen Mainza Shempela, Mwendalubi Albert Hadunka, Samson Mukale, Andrew Bambala, Malambo Mutila, Loveness Sakalimbwe, Fred Mulako Simwinji, Geoffrey Mainda, Chie Nakajima, Ruth Nakazwe, Fusya Goma, Nyambe Sinyange, Yasuhiko Suzuki, John Bwalya Muma, Roma Chilengi

**Affiliations:** 1 Antimicrobial Resistance Coordinating Committee, Zambia National Public Health Institute, Lusaka 10101, Zambia; zmuhimba@yahoo.com (Z.M.); clmusyami@yahoo.com (C.L.); or chichalesi2@gmail.com (C.C.); kasangaanita@gmail.com (M.K.); doreen.shempela@chaz.org.zm (D.M.S.); mukalesamson14@gmail.com (S.M.); lovesssakalimbwe@gmail.com (L.S.); ruthnakazwe@yahoo.com (R.N.); bsinyange@gmail.com (N.S.);; 2 Department of Pharmacy, University of Zambia, Lusaka 10101, Zambia; ckalungia@unza.ac.zm (A.C.K.); webrod.mufwambi@unza.ac.zm (W.M.); 3 Education and Continuous Professional Development Committee, Pharmaceutical Society of Zambia, Lusaka 10101, Zambia; 4 Department of Pathology, University Teaching Hospitals, Lusaka 10101, Zambia; 5 Centres for Infectious Disease Research in Zambia, Lusaka 10101, Zambia; amon.siame@cidrz.org (A.S.); bertha.chibwe@cidrz.org (B.C.); kaunda.kaunda@cidrz.org (K.K.); mwendalubi.hadunka@cidrz.org (M.A.H.); fred.simwinji@cidrz.org (F.M.S.); 6 Surveillance and Disease Intelligence Cluster, Zambia National Public Health Institute, Lusaka 10101, Zambia; mwangilwakelvin@yahoo.com (K.M.); priscillagardner82@gmail.com (P.G.); 7 Department of Pharmacy, Levy Mwanawasa Medical University, Lusaka 10101, Zambia; jimmyhangoma0282@gmail.com; 8 Department of Public Health, Copperbelt University, Lusaka 10101, Zambia; dakavictorm@gmail.com; 9 Department of Biomedical Sciences, School of Veterinary Medicine, University of Zambia, Lusaka 10101, Zambia; 10 Department of Clinical Studies, University of Zambia, Lusaka 10101, Zambia; ntombi.nkonde@unza.zm; 11 Department of Pathology and Microbiology, University Teaching Hospitals, Lusaka 10101, Zambia; 12 Hokudai Center for Zoonosis Control in Zambia, Hokkaido University, Lusaka 10101, Zambia; misheckshawa@czc.hokudai.ac.jp; 13 Division of International Research Promotion, International Institute for Zoonosis Control, Hokkaido University, Sapporo 001-0020, Japan; 14 Department of Veterinary Services, Ministry of Fisheries and Livestock, Lusaka 10101, Zambia; hermcham@gmail.com; 15 Department of Infectious Diseases, University Teaching Hospitals, Lusaka 10101, Zambia; duncanchanda@gmail.com; 16 Central Veterinary Research Institute, Ministry of Fisheries and Livestock, Lusaka 10101, Zambia; taonasinyawa@gmail.com (T.S.); fusyagoma@gmail.com (F.G.); 17 Action on Antibiotic Resistance (ReAct) Africa, Lusaka 10101, Zambia; kaundayamba@gmail.com; 18 Department of Health, World Health Organization, Lusaka 10101, Zambia; otipos@who.int (O.-T.S.); masaningaf@who.int (F.M.); mchibi@who.int (M.C.); mwadetsas@who.int (S.D.M.); 19 Department of Pharmacy, University Teaching Hospitals, Lusaka 10101, Zambia; bambalaandrew@gmail.com; 20 Food and Agriculture Organization of the United Nations, Lusaka 10101, Zambia; geoffrey.mainda@fao.org; 21 Division of Bioresources, Hokkaido University International Institute for Zoonosis Control, Sapporo 001-0020, Japansuzuki@czc.hokudai.ac.jp (Y.S.); 22 Division of Research Support, Hokkaido University Institute for Vaccine Research and Development, Sapporo 001-0020, Japan; 23 Department of Disease Control, University of Zambia, Lusaka 10101, Zambia; jmuma@unza.ac.zm

**Keywords:** antimicrobial resistance, antibiotics, bacteria, diagnostic stewardship, priority pathogens, sentinel surveillance, trends, Zambia

## Abstract

Background/Objectives: The right diagnostic tests, for the right patient, at the right time, are key to optimising antimicrobial use (AMU) and preventing antimicrobial resistance (AMR). This study evaluated diagnostic stewardship trends and AMR patterns in Zambian surveillance sentinel sites from 2020 to 2024. Methods: This descriptive, retrospective study analysed routine laboratory data from January 2020 to December 2024 at seven designated AMR surveillance sentinel sites across Zambia. Data on clinical specimens submitted for antimicrobial susceptibility testing were extracted from WHONET and analysed by year, specimen type, and antimicrobial susceptibility profile. Results: A total of 184,788 bacteriology specimens were processed over five years. Urine was the most frequently collected specimen, peaking above 20,000 in 2024. *Escherichia coli* was the most commonly isolated organism among 15 priority pathogens, comprising 25.9% of the 30,013 isolates. Though a statistically significant increasing trend in total organism isolations was observed annually, only *Shigella* sp. demonstrated a substantial increase in non-susceptibility to azithromycin (*p* = 0.027). High resistance was observed with doxycycline, azithromycin, clindamycin, trimethoprim/sulfamethoxazole, ciprofloxacin, and rifampicin, exhibiting resistance ranging from 50% to 80%. Critical AMR alerts included 65% for Vancomycin-Resistant *Enterococcus* (VRE), 72% for linezolid, and 44% for carbapenems, and possible ESBL-producing *Enterobacterales* showing the highest overall resistance at 35%, across sentinel sites. Conclusions: Zambia faces a concerning and significant increase in AMR, with high resistance prevalence across commonly used antibiotics. Critical resistance alerts for VRE, linezolid, carbapenems, and possible ESBL-producing *Enterobacterales* underscore an urgent need for robust antimicrobial stewardship and continuous diagnostic surveillance.

## 1. Introduction

Antimicrobial resistance (AMR) is a public health crisis of the 21st century, threatening to compromise the effective treatment of infections [1,2]. AMR has impacted the efficacy of commonly used antimicrobials, thereby drastically limiting treatment options and leaving few to no effective therapies for multidrug-resistant (MDR) or extensively drug-resistant (XDR) microorganisms reported across countries [2,3]. AMR is also associated with heightened morbidity and mortality rates, alongside substantial economic repercussions [2,4,5,6]. Projections indicate that, in the absence of effective mitigation strategies, AMR-related mortality may exceed 10 million annually with a cumulative economic cost of USD 100 trillion by 2050 [4,5]. Therefore, a multifaceted approach is imperative, encompassing robust surveillance systems, enhanced diagnostic stewardship (DS), and data-driven antimicrobial stewardship (AMS) interventions to curb this escalating public health threat [6]. DS and AMS are intrinsically linked [7,8]. DS relies on the judicious use of diagnostic tools to order the right tests for the right patient at the right time, and the findings inform the clinical diagnosis and rational use of antimicrobials [7,9,10]. In response to the AMR threat, Zambia has been implementing a multi-sectoral National Action Plan (NAP) for AMR (2017 to 2027) since 2017, with five key objectives aligned to the global action plan [11,12,13]. Among the Zambia NAP objectives are strengthening knowledge and evidence through surveillance and research, reducing the incidence of infection through effective sanitation, hygiene, and infection prevention measures and biosecurity, and optimising antimicrobial use across the health sector in a One Health (OH) approach [11,14]. This plan marked a significant step in initiating AMR surveillance and reporting to the World Health Organisation’s Global Antimicrobial Resistance and Use Surveillance System (GLASS), which harmonises global reporting on AMR and antimicrobial consumption (AMC) data [15,16].

Though steadily increasing, the capacity for AMR detection and DS in many low- and middle-income countries (LMICs), especially in Africa, is minimal [17,18,19], despite these being the cornerstones of AMR mitigation strategies, which enable the measurement of AMR burden and tracking the impact of interventions [20]. Due to resource constraints in Africa, DS is largely laboratory-based, generating phenotypic data on causative bacterial pathogens and their antibiotic susceptibility patterns, with very few laboratories able to conduct genomic AMR surveillance [20,21]. To address these gaps, regional initiatives such as WHO GLASS, the Africa CDC’s AMR Surveillance Network (AMRSNET), and the Fleming Fund have supported improvements in laboratory infrastructure, workforce training, and data harmonisation across the continent [19,22,23,24,25]. Additionally, efforts to enhance stakeholder collaboration across sectors are needed to ensure the efficient capture and continuous sharing of high-quality microbiology data for decision-making. Significant investments have been made in Zambia to enhance laboratory capacity through government initiatives and support from international partners such as the Fleming Fund and other global health development support [26,27]. Major challenges, however, remain, particularly in the development of sustainable digital infrastructure for AMR data management and reporting [18,28].

**Figure 1:**
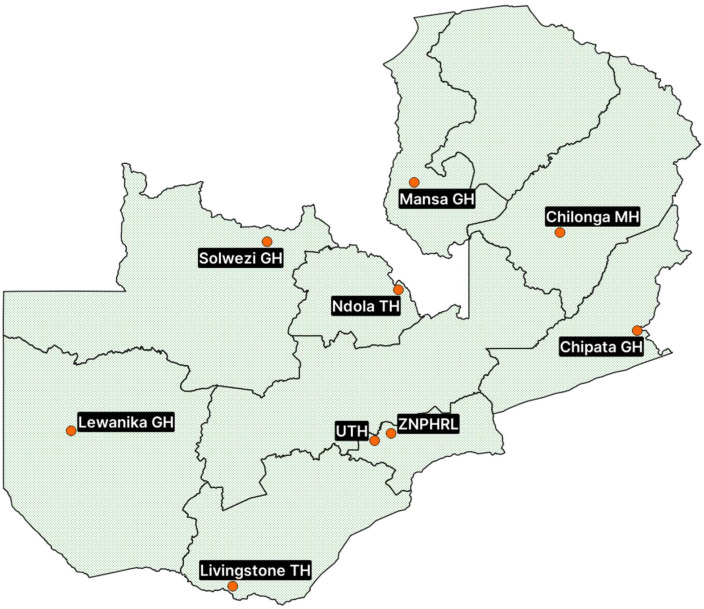
Map of Zambia indicating the location of the AMR surveillance sentinel sites for human health.

In Zambia, there is already evidence of drug-resistant clinical pathogens that have been isolated previously [14,29,30,31,32,33,34,35,36,37,38]. Additionally, there is evidence of inappropriate antibiotic use in the human health sector [39,40,41,42,43,44,45,46,47,48,49], especially those from the WHO ‘Watch’ group, despite AMS capacity-building interventions [50,51]. This underscores the need to establish and strengthen AMR surveillance across the country. The Antimicrobial Resistance Coordinating Committee (AMRCC) under the Zambia National Public Health Institute (ZNPHI) coordinates the implementation of the NAP. One of the objectives of the NAP is to strengthen surveillance and research. To this effect, eight AMR surveillance sentinel sites for human health (HH) have been established across Zambia (Figure 1). The sentinel sites forward-feed AMR surveillance data to the Zambia National Public Health Reference Laboratory (ZNPHRL). The sentinel sites have laboratory capacity to conduct Antimicrobial Susceptibility Tests (ASTs) and staff trained in diagnostic stewardship as part of the national AMR surveillance strategy. The AMR surveillance sentinel laboratories include the following: the University Teaching Hospitals (UTHs) in Lusaka province, Ndola Teaching Hospital (NTH) on the Copperbelt province, Livingstone University Teaching Hospital (LCH) in the Southern province, Chipata Central Hospital (CCH) in the Eastern province, Lewanika General Hospital (LGH) in the Western province, Mansa General Hospital (MGH) in Luapula province, Chilonga Mission Hospital (CMH) in Muchinga province, and Solwezi General Hospital (SGH) in the North-western province. The AMRCC has also established an additional five AMR surveillance sentinel sites for animal health (AH) across Zambia, as part of the national AMR surveillance framework [52].

Each sentinel HH surveillance laboratory in Zambia gathers AMR data from patients’ clinical specimens in the catchment population [52]. The surveillance laboratories are linked to WHONET, a microbiology laboratory database software employing a standardised approach to surveillance based solely on epidemiological, clinical, and population-based AMR data from blood, urine, stool, and cervical specimens [53,54,55]. Since the establishment of the AMR surveillance sentinel laboratories for HH in Zambia, following the first phase of the NAP implementation, information on the rate and extent of AMR detection, including the isolation rates of clinically important priority bacterial pathogens monitored globally, has not been published in Zambia to inform decision-making and public health actions. This study aimed to evaluate DS trends and AMR patterns in clinical pathogens isolated in the AMR surveillance sentinel sites in Zambia. The specific objectives were (i) to determine the types of bacteriology specimens collected and processed over the five years; (ii) to identify the most frequently isolated priority pathogens; and (iii) to assess the AMR patterns of isolated microorganisms, particularly for commonly used antimicrobials and critical resistance alerts. The findings of this study are critical in informing policy and decision-making to combat AMR.

## 2. Results

### 2.1. Bacteriology Specimens Analysed per Sentinel Site over Five Years

A total of 184,788 bacteriology specimens were collected, processed, and analysed over the five years. Table 1 presents the microbiological diagnostic testing capacity across the seven selected AMR surveillance laboratories over the five years. There was a statistically significant increase in the number of clinical specimens cultured for AST from January 2020 (baseline) to December 2024 (endpoint), with an absolute significant difference of 25,916 (*p* < 0.0001). Overall, all sentinel sites showed a statistically significant (*p* < 0.05) increase in the number of specimens cultured.

### 2.2. Specimen Collection Trends

**Figure 2:**
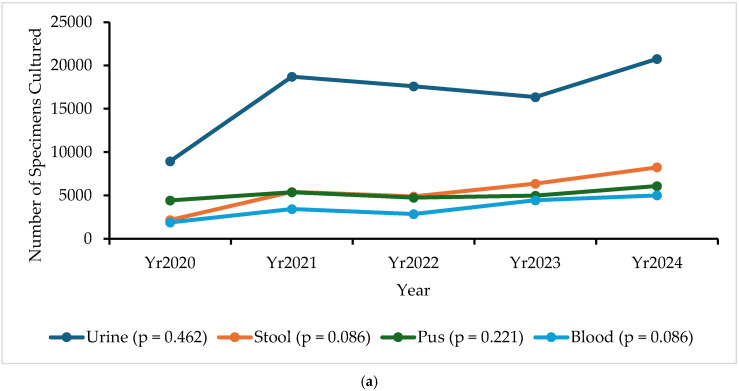

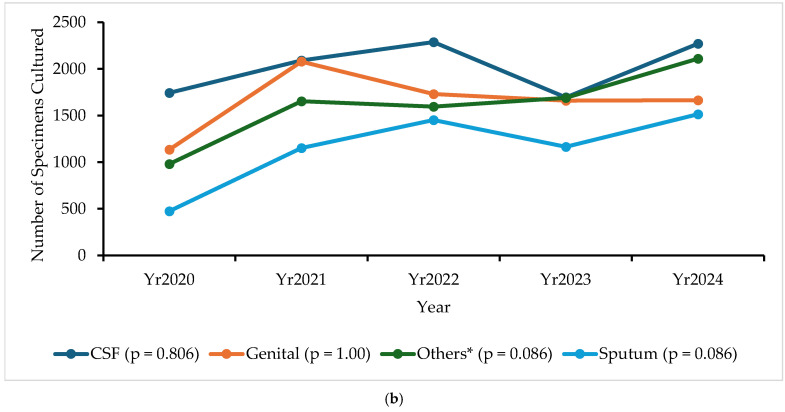
(**a**) Trends of specimens cultured in human AMR surveillance sentinel laboratories over five years (2020–2024). The line graph shows the specimen with relatively large numbers. The *p*-value represents the Sen’s slope for each specimen over time. (**b**). Number of specimens collected in human AMR surveillance sentinel laboratories by specimen types over five years (2020–2024). The line graph shows the specimens with relatively large numbers. The *p*-value represents the Sen’s slope for each specimen over time. * Other = soft tissue, biopsy, pleural fluid, peritoneal fluid, pericardial fluid, amniotic fluid, aspirate, autopsy, bile, and synovial fluid.

Figure 2a,b illustrate the collection trends of various specimen types across the participating sentinel surveillance laboratory facilities from 2020 to 2024. Urine consistently represents the most frequently collected specimen, peaking at over 20,000 specimens in 2024. Stool and pus specimens follow, with stool collection surpassing pus in later years, notably reaching 8244 specimens in 2024 compared to 6090 pus specimens collected in the same year. Blood specimen collection steadily increased over the period, while genital swab specimens remained relatively stable. Conversely, cerebrospinal fluid (CSF), sputum, and other specimens were consistently collected in much smaller quantities, with sputum being the least common. Overall, there was a clear upward trend in the number of specimens collected for most specimen types from 2020 to 2024, although the changes were not statistically significant (*p* > 0.05).

### 2.3. Isolation of Priority Pathogens

Table 2 shows the data for isolated organisms from 2020 to 2024, revealing several statistically significant trends. From the total of 30,013 isolates over the five years, *Escherichia coli* was the most frequently isolated organism, with a total of 7766 isolates, and showed a statistically significant increasing trend over the years (*p* = 0.003), rising from 825 isolates in 2020 to 2257 in 2024. *Staphylococcus aureus* also demonstrated a highly significant increasing trend (*p* = 0.0001), accumulating 4854 total isolates, with a mean of 971 isolates per year. *Klebsiella pneumoniae ss. pneumoniae* exhibited a statistically significant upward trend (*p* = 0.004), increasing from 455 isolates in 2020 to 1425 in 2024, for a total of 4517 isolates. Similarly, *Enterobacter* sp. (*p* < 0.0001), *Enterococcus* sp. (*p* = 0.005), and *Acinetobacter* sp. (*p* = 0.004) all showed statistically significant increasing trends in isolation numbers over the period. *Pseudomonas aeruginosa* also presented a statistically significant increasing trend (*p* = 0.001). Less common organisms like *Candida albicans* (*p* = 0.015), *Salmonella* sp. (*p* = 0.011), *Shigella* sp. (*p* = 0.012), *Streptococcus pneumoniae* (*p* = 0.009), *Neisseria gonorrhoeae* (*p* = 0.002), and *Haemophilus influenzae* (*p* = 0.022) also exhibit statistically significant increasing trends in their isolation rates. Notably, *Vibrio cholerae* (*p* = 0.275) and *Neisseria meningitidis* (*p* = 0.095) did not show statistically significant trends over the five years. The overall trend per year across all organisms was also statistically significant (*p*-values ranging from 0.004 to 0.009), indicating a general increasing trend in total organism isolations over the period.

### 2.4. Antimicrobial Susceptibility Patterns of Bacteria

Table 3 shows the antibiotic susceptibility patterns of bacteria measured as a percentage of the total number of isolates, including the WHO AWaRe classification. The highest resistance was demonstrated mainly by penicillin antibiotics (Oxacillin = 90%, ampicillin = 82%, and penicillin G = 82%). Cephalosporins showed intermediate resistance patterns, while carbapenems had the lowest proportion of resistance. Of the 33 antibiotics used, 15 were from the Access group, 14 were from the Watch group, and 4 were from the Reserve group (Table 3).

Notably, tigecycline, a Reserve group antibiotic, demonstrated 98% susceptibility among the 84 isolates tested, making it the most effective antibiotic across all the sites. Other antibiotics showing over 80% susceptibility included colistin (85%) and imipenem (81%), both from the Reserve group. Conversely, a significant number of antibiotics exhibited high resistance rates, suggesting diminished effectiveness. Penicillin (82%), doxycycline (52%), azithromycin (63%), tetracycline (57%), trimethoprim/sulfamethoxazole (74%), and ampicillin (82%) all showed resistance ranging from 50% to over 80%. Furthermore, spectinomycin, despite a small specimen size of 20 isolates, recorded an 80% resistance rate. The percentage of intermediate isolates to different antibiotics varied; however, linezolid, azithromycin, ciprofloxacin, and trimethoprim/sulfamethoxazole displayed noticeable percentages in the range of 10–20%, implying that dose adjustments or alternative treatments might be necessary for a substantial portion of infections requiring these drugs.

### 2.5. Important Antimicrobial Resistance (AMR) Alerts

Table 4 presents AMR alerts for key antibiotic classes identified among the isolates tested in the participating sentinel laboratories over the five years.

UTHs consistently reported the highest proportion of AMR cases for numerous categories, including 44% resistant cases for carbapenems, 72% for linezolid, and 65% cases of Vancomycin-resistant *Enterococcus*. Possible ESBL-producing *Enterobacterales* exhibited the highest overall resistance at 7809 (35%) of the cases, with significant contributions from UTHs (36%), NTH (15%), CCH (11%), MGH (23%), and LCH (11%). This was followed by fluoroquinolones at 7090 (32%), with UTHs accounting for 41% and MGH for 20% of the cases. Other substantial resistance categories include carbapenems (2326 resistant cases, with UTHs accounting for 44% and NTH 18%) and Methicillin-resistant *Staphylococcus aureus* (1263 resistant cases, with UTHs 57%, CCH 18%). MGH also stood out for penicillin resistance at 62%, while LCH showed a notable 38% decrease in susceptibility to meropenem. Conversely, categories like third-generation cephalosporin resistance and penicillin-resistant *Streptococcus pneumoniae* had relatively low total cases, and some hospitals, like LGH and CMH, generally showed lower percentages across most resistance categories.

### 2.6. Non-Susceptibility Trend Analysis Among Priority Pathogens

We analysed the non-susceptibility trends among the priority pathogens, which are under surveillance to determine the relative effectiveness of key antibiotics in clinical use and are key for AMS. Table 5 shows the findings of the trend analysis, indicating that generally, there were more decreasing than increasing non-susceptibility trends to key antibiotics among the priority pathogens tested, though the majority of trends were not statistically significant, with *Staphylococcus aureus* demonstrating a statistically significant decreasing trend in non-susceptibility to trimethoprim/sulfamethoxazole (*p* = 0.027). Only *Shigella* sp. demonstrated a statistically significant increasing trend in non-susceptibility to azithromycin (*p* = 0.027).

## 3. Discussion

This was the first such study to analyse the trends in diagnostic stewardship and AMR patterns across the Zambian AMR surveillance sentinel sites for human health over five years (2020 to 2024). We found several significant diagnostic surveillance and AMR trends, providing valuable insights into the epidemiology of bacterial infections and the evolving patterns of AMR, particularly among WHO priority pathogens. Over the five years, the progressive increase in bacteriology specimen volumes, especially in 2021, reflects improvements in diagnostic and reporting capabilities at participating sentinel sites and clinical practice culture. Given the large catchment population, the UTHs in Lusaka contributed over half of the total isolates, consistent with its role as the country’s main tertiary referral centre. Sites such as CCH, CMH, and LGH also showed notable contributions, indicating enhanced participation and improved microbiology capacities. Similar patterns of increased AST confirmations and reporting have been observed in other countries following the implementation of AMR surveillance programmes. For instance, Kenya documented increased AST rates following the integration of surveillance tools and training after the Fleming Fund support [56].

Urine was the most frequently collected specimen, followed by stool, pus, and blood. This pattern mirrors the findings in other African surveillance programmes where *Enterobacteriaceae*-causing UTIs are common reasons for AST [57,58,59,60]. *Escherichia coli* was the predominant uropathogen isolated, aligning with global and regional trends [61,62,63,64]. *Staphylococcus aureus* and Coagulase-Negative *Staphylococci* (CoNS) were frequently isolated from blood and wound specimens, consistent with their roles in skin, soft tissue, and bloodstream infections [65,66,67]. The isolation of commensal flora from a significant number of respiratory, genitourinary, and stool specimens suggests potential pre-analytical issues, such as poor specimen collection or transport delays, commonly seen in many settings, attributable to skills gaps and laboratory staff shortages [27,68,69]. This highlights the need for continuous training on specimen collection and internal quality control measures to ensure the reliability of surveillance data. Studies have also shown the importance of targeting diagnostic stewardship towards clinically relevant pathogens as a way of minimising the impact of normal flora, especially in specimens with normal flora [70,71].

Our findings offer critical insights into the AMR landscape in Zambia, with significant implications for clinical practice and public health within and beyond Zambia. Notwithstanding the limited number of isolates analysed, we found tigecycline (a Reserve antibiotic) standing out as an effective antimicrobial agent, demonstrating a 98% susceptibility rate. This suggests that tigecycline could be a valuable option for treating infections, particularly in cases where resistance to other antibiotics is prevalent. Similarly, the high susceptibility rates of isolated to carbapenems (with imipenem showing 81%, meropenem 79%, and ertapenem 78% susceptibility) underscore their continued importance in clinical use, considered as WHO Watch group antibiotics for severe bacterial infections [72,73,74,75,76,77,78]. The sustained effectiveness of the Watch and Reserve agents is crucial for managing serious healthcare-associated and multidrug-resistant infections [78,79,80], hence the need for enhanced AMS.

Conversely, the alarmingly high resistance observed in several commonly used antibiotics poses a substantial challenge. Penicillin G and ampicillin, both from the ‘Access’ group, exhibited a high resistance (82%) across the years (Table 2), similar to other settings [81]. This strongly implies that the empirical use of these antibiotics for suspected bacterial infections was largely ineffective, and their subsequent use should be re-evaluated. Such high resistance risks drive up AMR-associated mortality and costs, necessitating a shift towards alternative agents in subsequent treatment guidelines. Furthermore, the significantly increasing AMR rates for trimethoprim/sulfamethoxazole (74%) and azithromycin (63%) highlight the urgent need for local antibiograms to guide prescribing. With the well-established resistance patterns demonstrated, these antibiotics are less reliable for initial treatment, potentially leading to treatment failures, prolonged illness, and mortality. Intermediate susceptibility implies that higher doses or alternative antibiotics might be required to achieve therapeutic success, as standard dosages may not be sufficient to inhibit the pathogen. This necessitates careful consideration and therapeutic monitoring of pharmacokinetic and pharmacodynamic parameters of the patients when prescribing these agents, which, however, is not widely practicable in many resource-limited settings [82,83,84].

It was interesting to note that there were more decreasing than increasing non-susceptibility trends to key antibiotics among the priority pathogens tested over the five years, despite the majority of the trends being non-statistically significant. Our findings differ from those of a previous related study in Canada [85]. Observing more decreasing than increasing AMR trends is a cautiously positive epidemiological sign. It suggests that either specific antibiotics might be regaining or preserving efficacy or potentially validates AMS interventions. This finding should, however, be interpreted with significant caution as progress is often fragile, geographically heterogeneous, and pathogen-specific, potentially masking escalating AMR threats elsewhere. Moreover, methodological factors in the surveillance data necessitate scrutiny. Our AMR trend findings, while encouraging, underscore the imperative need for more sustained AMS, DS, and Infection Prevention Control (IPC) in Zambia to consolidate gains in mitigating AMR.

### 3.1. Implications for Policy and Practice

The policy and practice implications of the findings of this study are shown in Table 6. The findings of this study have several important policy and practice implications. Strengthening AMR surveillance systems and regularly updating local antibiograms are essential to guide empirical therapy in healthcare facilities. Regulatory enforcement of prescription-only antibiotic policies, alongside the integration of antimicrobial stewardship (AMS) programs across hospitals, pharmacies, and veterinary practices, is critical to curb misuse. Expanding laboratory diagnostic capacity and incorporating diagnostic stewardship into national health strategies will improve rational prescribing and reduce reliance on empirical treatment. Targeted AMR awareness campaigns and integration of AMR education into health professional curricula, combined with continuous professional development (CPD), are needed to improve knowledge among healthcare providers and the public. Furthermore, stricter control over the use of critically important antimicrobials (CIAs) in line with the WHO AWaRe framework should be prioritised to preserve their effectiveness. Finally, fostering One Health collaboration between the human, animal, and environmental sectors, while investing in behavioural and operational research, will ensure the development of context-specific interventions to strengthen AMR containment in Zambia and similar settings.

### 3.2. Study Limitations

Despite the valuable insights, this study had limitations. Firstly, we did not report the demographics of patients from where the specimens were collected, as these data were inconsistently recorded with incomplete patient metadata in the dataset, hindering detailed stratified analyses. Secondly, the reliance on phenotypic susceptibility data, without molecular and/or genomic data, limits our understanding of specific resistance mechanisms, such as ESBL and carbapenemase production. Thirdly, we relied on secondary data from WHONET and were unable to link clinical outcomes or treatment history, thereby limiting the evaluation of the clinical impact of resistance patterns. Finally, the inconsistent recording and incompleteness of patient metadata hindered detailed stratified analyses and the direct linking of AMR patterns to specific patient demographics or clinical outcomes. Notwithstanding this, our findings were robust and representative to guide future interventions.

## 4. Materials and Methods

### 4.1. Study Design and Setting

This retrospective study analysed routine AMR laboratory data from January 2020 to December 2024 collected from seven designated AMR surveillance sentinel sites across Zambia. These sentinel sites included the University Teaching Hospitals (UTHs), Ndola Teaching Hospital (NTH), Livingstone Teaching Hospital (LTH), Chipata Central Hospital (CCH), Lewanika General Hospital (LGH), Mansa General Hospital (MGH), and Chilonga Mission Hospital (CMH). The selection of these facilities was based on their geographic distribution, diagnostic capacity, case volume, and active participation in the Zambia Integrated Antimicrobial Resistance Surveillance Framework. These sentinel sites represent a mix of urban and rural referral hospitals, each serving diverse catchment populations with varied demographics and disease profiles of the patients. The inclusion of both tertiary referral centres (such as UTH and NTH) and provincial-level hospitals enables the surveillance system to capture a broader spectrum of bacterial pathogens and antimicrobial resistance patterns observed in both inpatient and outpatient settings.

The study period was selected to capture trends before, during, and after the COVID-19 pandemic, which has been shown to influence antibiotic usage and AMR patterns globally [43,86]. We chose this study period to encompass the pandemic and allow for observation of potential trends, acknowledging the global context while being transparent about the absence of specific local correlative data within our dataset. The selected facilities collectively serve an estimated 40–60% of Zambia’s provincial referral healthcare populations, ensuring national representation of AMR trends. This sentinel surveillance network forms a core component of Zambia’s One Health strategy to combat AMR across human, animal, and environmental sectors. Each site submitted monthly WHONET exports, which were validated for completeness and de-duplicated centrally at ZNPHRL. All AMR surveillance sentinel sites have been supported by a mentorship programme to ensure standardised laboratory specimen analysis and reporting.

This study included all patients, both inpatients and outpatients, who presented to the sentinel health facilities and had clinical specimens collected for AST. Eligible specimens comprised bacterial isolates from blood, urine, stool, respiratory tract, genital swabs, soft tissues, and other body fluids [87,88,89]. Only the first isolate per patient per infection episode (defined as within 14 days from the same anatomical site) was included to prevent overestimation of resistance rates [90]. Isolates with incomplete AST results or collected outside the defined study period were excluded.

### 4.2. Specimen Collection and Microbiological Procedures

Clinical specimens were collected by trained healthcare personnel following standard clinical protocols previously described [91,92]. Upon receipt in the microbiology laboratories, specimens were cultured using appropriate selective and non-selective media, such as blood agar, MacConkey agar, chocolate agar, and cystine–lactose–electrolyte-deficient (CLED) agar, depending on the type of specimen. Specimens collected at peripheral units were transported within 24–48 h using cold-chain protocols (2–8 °C) where feasible, ensuring specimen viability. Incubation was performed at 37 °C for 18–24 h. Initial bacterial classification was conducted using Gram staining, followed by identification through conventional biochemical tests, catalase and coagulase tests, and, where available, automated identification systems, such as the Vitek^®^ 2 Compact (bioMérieux, France) were used to support accurate and rapid identification. All laboratory procedures adhered to national biosafety guidelines, with Category A waste disposed of according to the WHO-recommended protocols.

### 4.3. Antimicrobial Susceptibility Testing (AST)

AST was performed using the Kirby–Bauer disk diffusion, minimum inhibitory concentration (MIC) methods [93,94] and/or automated methods, such as the Vitek^®^ 2 system, depending on each facility’s capacity. Interpretations of inhibition zones (disk diffusion) and minimum inhibitory concentrations (MICs) were conducted following Clinical and Laboratory Standards Institute (CLSI) guidelines applicable during each year of the study. AST panels were stratified based on infection site (e.g., bloodstream, urinary, respiratory) to ensure clinical relevance of susceptibility testing. Laboratories conducted routine internal quality assurance (IQA) on all laboratory processes and were enrolled in external quality assessment (EQA) programs supported by the WHO and the African Society for Laboratory Medicine (ASLM). Due to intermittent stock-outs of reagents in 2021, some antibiotics were not uniformly tested across all sites, which may influence longitudinal comparability.

The antimicrobial susceptibility of isolates was tested against a panel of antibiotics as recommended and following the Clinical Laboratory Standards Institute (CLSI), including but not limited to:

- β-lactams: ampicillin (AMP), Amoxicillin–Clavulanate (AMC), ceftriaxone (CRO), cefotaxime (CTX), cefepime (FEP), Ceftazidime (CAZ), cefuroxime (CXM), cefazolin (CZ), and cefoxitin (FOX);
- Carbapenems: imipenem (IPM), meropenem (MEM), and doripenem (DOR);
- Aminoglycosides: gentamicin (GEN), tobramycin (TOB), and amikacin (AK);
- Fluoroquinolones: ciprofloxacin (CIP), levofloxacin (LEV), ofloxacin (OFX), norfloxacin (NOR), and nalidixic acid (NA);
- Macrolides and others: erythromycin (ERY), azithromycin (AZM), clarithromycin (CLA), clindamycin (CLI), linezolid (LZD), vancomycin (VAN), doxycycline (DOX), tetracycline (TET), Chloramphenicol (CHL), nitrofurantoin (NIT), trimethoprim-sulfamethoxazole (SXT), and Quinupristin/Dalfopristin (RP).

Quality control was performed using American Type Culture Collection (ATCC) reference strains, such as *Escherichia coli* ATCC 25922, *Staphylococcus aureus* ATCC 25923, and *Pseudomonas aeruginosa* ATCC 27853 [15,94]. Resistance profiles were categorised as susceptible (S), intermediate (I), or resistant (R). Multidrug-resistant (MDR) organisms were defined as isolates non-susceptible to at least one agent in three or more antimicrobial categories, as per the international consensus guidelines [89].

**Figure 3:**
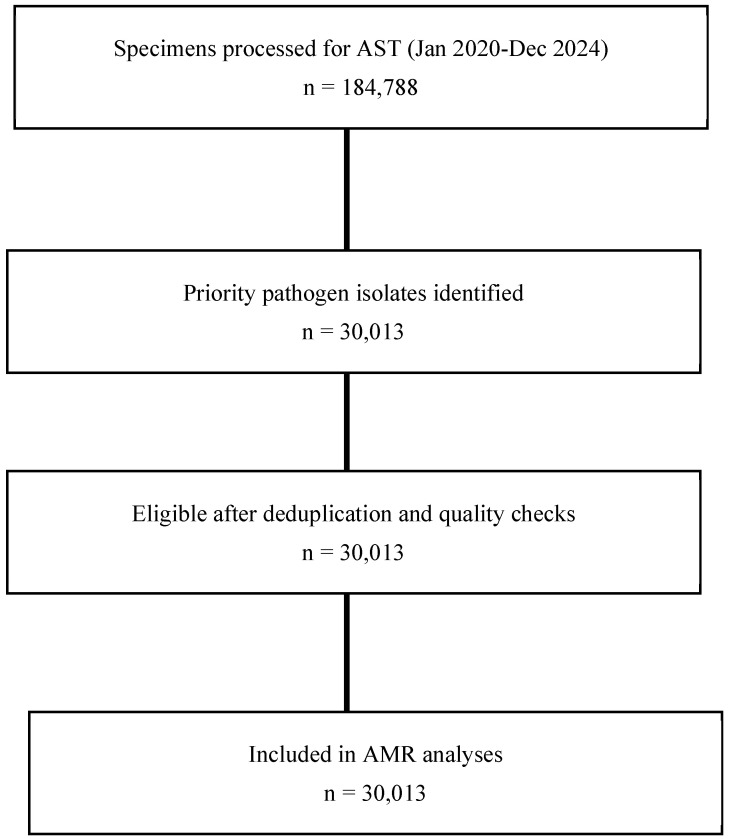
Flow diagram built from manuscript data. Totals: specimens processed = 184,788; priority pathogen isolates = 30,013.

The flow diagram of the methodology procedure and dataset selection for AMR analyses (January 2020–December 2024) is shown in Figure 3.

### 4.4. Data Management

Microbiological and AST data were captured electronically over the study period using WHONET 2025 software (WHO, Geneva), enabling standardised data entry across all sentinel sites. Data were cleaned, validated, and deduplicated by site coordinators and then consolidated into a central national database managed by the ZNPHRL. Isolates were deduplicated based on patient ID, specimen collection date, and pathogen to ensure a single entry per infection episode. Patient demographics (age, sex), clinical information (specimen type, inpatient/outpatient status), and microbiological results (pathogen, AST profile) were included. Bacteria were grouped by Gram stain, species, and classified according to the WHO GLASS priority pathogen list.

### 4.5. Data Analysis

Descriptive statistics were performed using WHONET 2025 and XLSTAT 2025 (Lumivero, Denver, CO, USA). Analyses included frequencies, proportions, and resistance rates, stratified by year, site, specimen type, and pathogen. AMR patterns were reviewed over the five years (2020–2024), and data were visualised using charts and tables. AMR rates were reported as a percentage of resistant isolates per total tested for each antibiotic, in line with the established surveillance and reporting standards [15,95]. DS trends for priority pathogens were analysed using the Cochran–Armitage test for trend, with attention to resistance alerts against critical antibiotics listed in the WHO Watch and Reserve groups. Statistical significance was accepted at a 95% confidence interval. We used the Mann–Kendall test to assess whether AMR among priority pathogens was statistically significantly increasing or decreasing over time. A Sen’s slope (S) value greater than 0 indicated an upward trend, while an S value less than 0 indicated a downward trend.

## 5. Conclusions

There was a substantial rise in bacteriology specimen processing capacity from 2020 to 2024 in the seven selected AMR sentinel sites. Urine consistently represented the most frequently collected specimen type, demonstrating a peak of over 20,000 specimens in 2024, followed by pus and stool specimens. *Escherichia coli* was the most isolated priority pathogen. A statistically significant increasing trend in the overall number of isolated organisms was observed annually, indicating a general improvement in diagnostic stewardship over the five years. Although we found more decreasing than increasing non-susceptibility trends to key antibiotics among the priority pathogens tested, a concerning prevalence of high AMR was evident across several commonly used antimicrobial agents. Critically, high AMR alerts were noted for Vancomycin-resistant *Enterococcus*, linezolid, and carbapenems. These findings underscore an urgent need for robust diagnostic stewards and antimicrobial stewardship programs alongside continuous AMR surveillance in Zambia.

## Figures and Tables

**Table 1:** Total number of bacteriology specimens analysed by the sentinel site over 5 years (from 2020 to 2024).

Sentinel Site	Total Specimens Cultured, n (%)					Total
	2020	2021	2022	2023	2024	
UTHs	11,719 (53.8)	23,329 (58.4)	19,335 (52.1)	16,265 (42.4)	21,798 (45.7)	92,443
NTH	4673 (21.5)	5449 (13.7)	5504 (14.2)	5988 (15.6)	8296 (17.4)	29,910
LCH	2556 (11.7)	4401 (11.0)	4937 (13.3)	5256 (13.7)	5442 (11.4)	22,592
MGH	1253 (5.8)	2601 (6.5)	3070 (8.3)	3618 (9.4)	4310 (9.0)	14,852
CCH	840 (3.9)	2535 (6.4)	2996 (8.1)	3242 (8.5)	3138 (6.6)	12,751
CMH	633 (2.9)	794 (2.0)	651 (1.8)	2454 (6.4)	3039 (6.4)	7571
LGH	82 (0.4)	793 (2.0)	636 (1.7)	1509 (3.9)	1649 (3.5)	4669
Grand Total	21,756 (100)	39,899 (100)	37,129 (100)	38,332 (100)	47,672 (100)	184,788

Abbreviations: UTHs = The University Teaching Hospitals; NTH = Ndola Teaching Hospital; LCH = Livingstone University Teaching Hospital; CCH = Chipata Central Hospital; LGH = Lewanika General Hospital; MGH = Mansa General Hospital; CMH = Chilonga Mission Hospital.

**Table 2:** Diagnostic stewardship trends in organism isolation from January 2020 to December 2024.

Organisms	Total	Period By Years (2020 to 2024)					*p*-Value of Trend per Organism
		Year 2020	Year 2021	Year 2022	Year 2023	Year 2024	
*Escherichia coli*	7766	825	1275	1638	1771	2257	0.003
*Staphylococcus aureus*	4854	754	1111	924	982	1083	0.0001
*Klebsiella pneumoniae*	4517	455	819	855	963	1425	0.004
*Enterobacter* sp.	4409	598	977	1008	774	1052	<0.0001
*Enterococcus* sp.	2790	232	501	595	609	853	0.005
*Acinetobacter* sp.	1655	178	296	303	359	519	0.004
*Pseudomonas aeruginosa*	1443	243	340	246	232	382	0.001
*Candida albicans*	723	107	157	71	113	275	0.015
*Salmonella* sp.	564	30	131	84	155	164	0.011
*Vibrio cholerae*	537			9	117	411	0.275
*Shigella* sp.	395	28	70	70	87	140	0.012
*Streptococcus pneumoniae*	237	27	42	33	52	83	0.009
*Neisseria gonorrhoeae*	85	15	25	14	20	11	0.002
*Haemophilus influenzae*	29	8	1	3	8	9	0.022
*Neisseria meningitidis*	9		4		4	1	0.095

**Table 3:** Antimicrobial susceptibility patterns of the bacteria across the AMR sentinel sites (January 2020 to December 2024). The antibiotics are sorted by resistance level from highest to lowest.

Antibiotic Name	WHO AWaRe Classification	No. of Isolates	%R	%I	%S
Oxacillin	Access	484	90%	0%	10%
Ampicillin	Access	10,989	82%	4%	14%
Penicillin G	Access	8756	82%	0%	17%
Spectinomycin	Access	20	80%	10%	10%
Cefazolin	Access	4566	77%	9%	14%
Trimethoprim/Sulfamethoxazole	Access	11,362	74%	3%	23%
Trimethoprim	Access	87	72%	3%	24%
Azithromycin	Watch	3879	63%	4%	33%
Nalidixic acid	Access	6395	62%	7%	31%
Amoxicillin/Clavulanic acid	Access	7385	61%	14%	26%
Cefotaxime	Watch	6922	59%	5%	36%
Tetracycline	Access	14,913	57%	7%	37%
Ceftriaxone	Watch	7822	57%	5%	39%
Ceftazidime	Watch	8087	56%	9%	35%
Cefoxitin	Watch	6601	55%	0%	44%
Cefepime	Watch	7757	53%	4%	43%
Amoxicillin	Access	312	53%	13%	35%
Doxycycline	Access	2585	52%	8%	40%
Erythromycin	Access	10,388	49%	17%	34%
Quinupristin/Dalfopristin	Reserve	1335	46%	3%	51%
Ciprofloxacin	Watch	19,094	45%	11%	44%
Norfloxacin	Watch	2449	44%	6%	50%
Gentamicin	Access	17,000	40%	9%	50%
Vancomycin	Watch	2944	38%	11%	51%
Clindamycin	Watch	6132	31%	15%	54%
Rifampicin	Watch	571	31%	2%	67%
Chloramphenicol	Access	12,983	23%	6%	70%
Linezolid	Reserve	5081	19%	11%	71%
Ertapenem	Watch	3098	15%	7%	78%
Colistin	Reserve	162	15%	0%	85%
Meropenem	Watch	6907	13%	8%	79%
Imipenem	Watch	7104	11%	8%	81%
Tigecycline	Reserve	84	2%	0%	98%

**Table 4:** Important AMR alerts for key antibiotic classes detected by sentinel surveillance sites over 5 years (January 2020 to December 2024).

Important Resistance Alerts	UTH	LGH	NTH	CCH	MGH	CMH	LCH	Total
	n (%)	n (%)	n (%)	n (%)	n (%)	n (%)	n (%)	
Carbapenems = non-susceptible	1018 (44)	93 (4)	418 (18)	166 (7)	250 (11)	35 (2)	346 (15)	2326
Cephalosporin III = non-susceptible	32 (28)	1 (1)	18 (16)	3 (3)	42 (37)	3 (3)	15 (13)	114
Fluoroquinolones = non-susceptible	2896 (41)	145 (2)	170 (3)	1200 (17)	1449 (20)	318 (4)	912 (13)	7090
Imipenem = Possible decreased susceptibility	167 (46)	2 (1)	110 (30)	16 (4)	48 (13)	8 (2)	14 (4)	365
Linezolid = non-susceptible	941 (72)	60 (5)	41 (3)	45 (3)	61 (5)	61 (5)	89 (7)	1298
Meropenem = Possible decreased susceptibility	87 (19)	21 (5)	56 (13)	44 (10)	57 (13)	13 (3)	170 (38)	448
Methicillin-resistant Staphylococcus aureus	726 (57)	59 (5)	9 (1)	231 (18)	117 (9)	51 (4)	70 (6)	1263
Oxacillin = Tested by disk diffusion	144 (61)	15 (6)	-	-	48 (20)	12 (5)	47 (20)	236
Penicillin-non-susceptible S. pneumoniae	4 (57)	1 (14)	-	-	2 (29)	-	-	7
Penicillins = non-susceptible	23 (11)	4 (2)	22 (11)	11 (5)	127 (62)	10 (5)	9 (4)	206
Possible ESBL-producing Enterobacterales	2826 (36)	140 (2)	1204 (15)	821 (11)	1802 (23)	161 (2)	855 (11)	7809
Quinupristin/Dalfopristin = non-susceptible	51 (60)	5 (6)	-	29 (34)	-	-	-	85
Vancomycin or Teicoplanin = non-susceptible	92 (64)	-	22 (15)	11 (8)	12 (8)	-	7 (5)	144
Vancomycin or Teicoplanin = Non-susceptible (Disk diffusion)	24 (100)	-	-	-	-	-	-	24
Vancomycin-resistant Enterococcus	505 (65)	2 (0.3)	82 (11)	74 (9)	38 (5)	7 (1)	72 (9)	780
Grand Total	11,340 (51)	603 (3)	2873 (13)	3065 (14)	4487 (20)	788 (4)	2907 (13)	22,195

UTHs = University Teaching Hospitals; LGH = Lewanika General Hospital; NTH = Ndola Teaching Hospital; CCH = Chipata Central Hospital; MGH = Mansa General Hospital; CMH = Chilonga Mission Hospital; LCH = Livingstone University Teaching Hospital.

**Table 5:** Summary of observed trends in non-susceptible priority bacteria isolates from AMR sentinel surveillance sites (January 2020 to December 2024).

Bacteria Isolate and Priority Antibiotics Tested	Proportion (%) of Non-Susceptible Isolates by Year					Results of the Mann-Kendall Test		
	2020	2021	2022	2023	2024	Kendall’s Tau	*p*-Value	Sen’s Slope
*Escherichia coli* (*n* = 8052)	864	1299	1645	1777	2467			
CIP (*W*)	61.9	67.1	68.4	56.2	66.3	0.000	1.000	0.417
FEP (*W*)	45.4	52.2	58.6	47.5	49.9	0.200	0.806	0.913
CTX (*W*)	73.3	62.4	60.2	58.0	62.4	−0.527	0.312	−2.200
CRO (*W*)	60.0	70.0	53.0	48.3	57.6	−0.400	0.462	−3.700
IPM (*W*)	9.2	8.4	4.6	10.8	10.0	0.200	0.806	0.367
MEM (*W*)	9.7	20.5	12.2	32.6	8.8	0.000	1.000	0.513
SXT (*A*)	82.7	88.9	91.8	78.4	79.6	−0.200	0.806	−1.104
NIT (*A*)	30.9	21.6	19.8	31.3	24.0	0.000	1.000	−0.796
*Klebsiella pneumoniae* (*n* = 4647)	469	834	856	971	1517			
CIP (*W*)	70.7	77.3	65.4	70.9	64.8	−0.400	0.462	−2.063
FEP (*W*)	68.6	62.9	73.2	77.0	64.0	0.200	0.806	1.333
CTX (*W*)	75.5	86.8	74.2	78.6	74.8	−0.200	0.806	−0.413
CRO (*W*)	75.8	85.7	73.6	70.6	74.4	−0.400	0.462	−1.417
IPM (*W*)	21.6	23.3	9.5	11.7	16.8	−0.200	0.806	−1.683
MEM (*W*)	33.3	29.1	20.4	39.9	14.7	−0.400	0.462	−4.425
SXT (*A*)	78.7	88.8	85.7	78.6	80.1	−0.200	0.806	−1.417
NIT (*A*)	68.8	51.6	49.8	59.0	55.5	−0.200	0.806	−2.533
*Acinetobacter* sp. (n = 1709)	179	297	305	364	564			
CIP (*W*)	57.6	58.0	56.5	54.7	53.5	−0.800	0.086	−1.350
FEP (*W*)	74.2	57.4	53.8	51.6	60.2	−0.400	0.462	−3.200
CTX (*W*)	83.3	70.4	75.9	81.7	77.8	0.000	1.000	0.208
CRO (*W*)	70.0	79.4	63.0	83.3	78.9	0.200	0.806	2.088
CAZ (*W*)	70.9	74.3	76.1	76.4	65.0	0.200	0.806	0.675
GEN (*A*)	53.4	52.6	38.0	52.1	45.5	−0.600	0.221	−1.388
IPM (*W*)	31.4	19.5	18.9	25.0	20.0	−0.200	0.806	−1.367
MEM (*W*)	0.0	26.1	25.0	42.6	28.2	0.600	0.221	7.650
TCY (*A*)	71.4	65.6	30.0	47.3	55.3	−0.400	0.462	−4.913
SXT (*A*)	83.8	74.8	71.4	57.5	65.7	−0.800	0.086	−5.363
*Pseudomonas aeruginosa* (*n* = 1488)	253	343	247	238	407			
CIP (*W*)	24.2	28.3	18.6	21.5	25.6	0.000	1.000	−0.275
FEP (*W*)	30.4	41.3	29.2	29.3	35.9	0.000	1.000	−0.133
CAZ (*W*)	56.6	35.0	45.7	47.5	29.7	−0.400	0.462	−4.242
GEN (*A*)	45.4	38.1	35.3	18.0	34.8	−0.800	0.086	−3.925
IPM (*W*)	11.6	9.3	0.0	15.0	9.6	0.000	1.000	−0.200
MEM (*W*)	7.7	21.1	28.6	25.0	17.3	0.200	0.806	2.175
AMK (*A*)	0.0	18.4	8.3	16.2	12.9	0.200	0.806	2.763
TZP (*W*)	49.3	36.0	21.4	18.2	26.0	−0.600	0.221	−7.363
*Salmonella* sp. (*n* = 547)	30	119	73	156	169			
AMP (*A*)	100.0	62.3	70.6	67.4	56.3	−0.600	0.221	−9.008
AZM (*W*)	0.0	5.3	0.0	7.1	0.0	0.120	1.000	0.000
CIP (*W*)	100.0	66.7	40.5	37.5	57.9	−0.600	0.221	−12.563
CTX (*W*)	50.0	16.0	14.3	10.7	25.6	−0.400	0.462	−3.125
CRO (*W*)	33.3	20.0	25.0	16.0	9.2	−0.800	0.086	−5.896
IPM (*W*)	0.0	7.4	5.0	9.1	21.1	0.800	0.086	4.333
TCY (*A*)	60.0	22.4	60.0	35.8	40.0	−0.105	1.000	−2.500
SXT (*A*)	57.1	60.0	45.0	53.3	49.2	−0.400	0.462	−2.663
*Shigella* sp. (*n* = 401)	30	70	70	88	143			
AMP (*A*)	69.2	55.6	60.7	76.0	81.0	0.600	0.221	5.050
AZM (*W*)	0.0	22.9	24.1	35.7	37.0	1.000	0.027 *	7.850
CIP (*W*)	25.0	28.9	32.4	30.8	28.0	0.200	0.806	0.850
CTX (*W*)	0.0	19.2	11.1	8.3	11.5	0.200	0.806	1.483
CRO (*W*)	50.0	6.7	12.5	7.4	8.5	−0.200	0.806	−3.550
IPM (*W*)	0.0	16.7	10.5	16.7	6.7	0.105	1.000	0.838
TCY (*A*)	90.9	76.7	53.8	82.4	76.7	−0.316	0.613	−3.192
SXT (*A*)	75.0	85.2	100.0	88.2	85.7	0.400	0.462	2.088
*Enterobacter* sp. (*n* = 4481)	615	982	1019	776	1089			
CIP (*W*)	63.6	60.2	57.4	65.1	65.4	0.400	0.462	0.475
FEP (*W*)	50.5	51.9	47.3	58.1	47.3	−0.105	1.000	−0.400
CTX (*W*)	56.9	66.3	67.0	70.3	65.2	0.400	0.462	2.038
CRO (*W*)	70.5	65.6	59.4	62.3	66.3	−0.200	0.806	−1.350
CAZ (*W*)	69.8	64.3	65.2	59.0	68.3	−0.200	0.806	−1.338
IPM (*W*)	17.7	21.0	13.2	13.1	16.5	−0.400	0.462	−0.900
MEM (*W*)	6.7	18.1	12.7	33.1	14.8	0.400	0.462	2.513
TCY (*A*)	65.5	66.1	79.5	62.7	68.8	0.200	0.806	0.712
SXT (*A*)	77.6	82.1	81.9	74.4	77.9	−0.200	0.806	−0.633
NIT (*A*)	40.7	29.5	26.3	34.6	36.2	0.000	1.000	0.238
*Staphylococcus aureus* (*n* = 4984)	781	1117	928	1005	1153			
PEN (*A*)	93.2	91.4	90.4	93.5	93.0	0.000	1.000	0.025
CIP (*W*)	39.3	41.9	35.4	29.7	36.5	−0.400	0.462	−1.875
FOX (*W*)	42.5	44.4	33.2	37.1	39.6	−0.200	0.806	−1.163
LNZ (*R*)	48.9	45.5	17.1	20.5	38.3	−0.400	0.462	−3.025
TCY (*A*)	36.4	44.2	48.7	30	28.8	−0.400	0.462	−2.017
SXT (*A*)	73.4	67.1	58.7	54.3	42.5	−1.000	0.027 *	−7.538
NIT (*A*)	20.8	7.8	14.3	3.8	6.5	−0.600	0.221	−3.413
*Enterococcus* sp. (*n* = 2844)	237	504	600	614	889			
AMP (*A*)	44.6	40.1	48.5	32.9	32.7	−0.600	0.221	−3.288
PEN (*A*)	42.5	47.5	34.1	54.4	51.9	0.400	0.462	2.900
CIP (*W*)	62.9	67.9	74.8	58.5	64.8	0.000	1.000	−0.279
LNZ (*R*)	31.0	33.1	19.6	6.4	27.3	−0.400	0.462	−3.817
TCY (*A*)	61.7	79.0	86.0	71.7	77.0	0.200	0.806	3.579
VAN (*W*)	45.6	50.0	59.0	55.9	46.0	0.200	0.806	1.525
NIT (*A*)	45.5	18.3	28.1	34.1	26.1	−0.200	0.806	−2.400

Abbreviations: AMC = Amoxicillin/Clavulanic acid; AMK = amikacin; AMP = ampicillin; AMX = Amoxicillin; AZM = azithromycin; CAZ = Ceftazidime; CHL = Chloramphenicol; CIP = ciprofloxacin; CLI = clindamycin; COL = colistin; CRO = ceftriaxone; CTX = cefotaxime; CZO = cefazolin; DOX = doxycycline; ERY = erythromycin; ETP = ertapenem; FEP = cefepime; FOX = cefoxitin; GEN = gentamicin; IPM = imipenem; LNZ = linezolid; MEM = meropenem; NAL = nalidixic acid; NIT = nitrofurantoin; NOR = norfloxacin; OXA = Oxacillin; PEN = penicillin G; QDA = Quinupristin/Dalfopristin; RIF = Rifampicin; SPT = spectinomycin; SXT = trimethoprim/sulfamethoxazole; TCY = tetracycline; TMP = trimethoprim; TGC = tigecycline; TZP = Piperacillin/Tazobactam; VAN = Vancomycin; (*A*) = Access; (*W*) = Watch; (*R*) = Reserve. * Statistically significant.

**Table 6:** Policy and practice implications for strengthening AMR containment.

Key Finding	Policy Implication	Practice Recommendation
High levels of antibiotic resistance observed across key bacterial pathogens	Strengthen national AMR surveillance systems to ensure continuous monitoring and reporting	Regular updates of local antibiograms to guide empirical therapy in hospitals and community health facilities
Overuse and inappropriate prescribing of antibiotics in both the human and veterinary health sectors	Enforce stricter prescription-only policies for antibiotics and regulate sales in pharmacies and agro-veterinary outlets	Implement AMS programs across hospitals, community pharmacies, and veterinary practices
Limited laboratory diagnostic capacity leading to empirical prescribing	Prioritise investment in diagnostic infrastructure and integrate diagnostic stewardship into national health policies	Expand access to culture and sensitivity testing to support rational prescribing
Inadequate awareness of AMR among healthcare providers and the public	Develop targeted AMR awareness campaigns and integrate AMR education into professional training curricula	Conduct continuous professional development (CPD) for healthcare workers and community sensitisation programs
Use of critically important antimicrobials (CIAs) without appropriate restrictions	Adopt and enforce national treatment guidelines aligned with the WHO AWaRe classification	Restrict the use of Reserve and Watch group antibiotics to cases with proven sensitivity or specialist approval
Weak inter-sectoral collaboration (human, animal, and environmental health) in AMR containment	Strengthen the One Health AMR coordination framework and harmonise policies across sectors	Promote joint human-animal health surveillance, training, and public awareness campaigns
Lack of research into behavioural and economic drivers of irrational antibiotic use	Support operational and behavioural research to inform AMR policies	Engage communities and healthcare providers in designing context-specific AMS interventions

## Data Availability

Research data used in this study are available upon reasonable request from the Zambia National Public Health Institute (ZNPHI).

## Abbreviations

The following abbreviations are used in this manuscript:

AMC	Amoxicillin/Clavulanic acid
AMP	Ampicillin
AMR	Antimicrobial resistance
AMS	Antimicrobial stewardship
AMX	Amoxicillin
AZM	Azithromycin
CAZ	Ceftazidime
CCH	Chipata Central Hospital
CHL	Chloramphenicol
CIP	Ciprofloxacin
CLI	Clindamycin
CMH	Chilonga Mission Hospital
COL	Colistin
COVID-19	Corona Virus Disease 2019
CRO	Ceftriaxone
CSF	Cerebrospinal fluid
CTX	Cefotaxime
CZO	Cefazolin
DOX	Doxycycline
DS	Diagnostic stewardship
ERY	Erythromycin
ETP	Ertapenem
FEP	Cefepime
FOX	Cefoxitin
GEN	Gentamicin
GLASS	Global Antimicrobial Resistance and Use Surveillance System
IMP	Imipenem
LUTHLUTH	Livingstone University Teaching Hospital
LGH	Lewanika General Hospital
LNZ	Linezolid
MEM	Meropenem
MGH	Mansa General Hospital
NAL	Nalidixic acid
NAP	National Action Plan
NIT	Nitrofurantoin
NOR	Norfloxacin
NTH	Ndola Teaching Hospital
OXA	Oxacillin
PEN	Penicillin G
QDA	Quinupristin/Dalfopristin
RIF	Rifampicin
SPT	Spectinomycin
SXT	Trimethoprim/Sulfamethoxazole
TCY	Tetracycline
TGC	Tigecycline
TMP	Trimethoprim
UTHs	University Teaching Hospitals
VAN	Vancomycin
ZNPHI	Zambia National Public Health Institute
